# Spectral Embedded Deep Clustering

**DOI:** 10.3390/e21080795

**Published:** 2019-08-15

**Authors:** Yuichiro Wada, Shugo Miyamoto, Takumi Nakagama, Léo Andéol, Wataru Kumagai, Takafumi Kanamori

**Affiliations:** 1 Graduate School of Information Science, Nagoya University, Furo-cho, Chikusa-ku, Nagoya 464-8601, Japan; 2 Department of Systems Innovation, School of Engineering, The University of Tokyo, Hongo Campus, Eng. Bldg. No. 3, 2F, 7-3-1 Hongo, Bunkyo-ku, Tokyo 113-8656, Japan; 3 Department of Mathematical and Computing Science, School of Computing, Tokyo Institute of Technology, 2-12-1 Ookayama, Meguro-ku, Tokyo 152-8552, Japan; 4 Computer Science Department, Sorbonne Université, 4 place Jussieu, 75005 Paris, France; 5 RIKEN AIP, Nihonbashi 1-chome Mitsui Building, 15th floor, 1-4-1 Nihonbashi, Chuo-ku, Tokyo 103-0027, Japan

**Keywords:** clustering, deep neural networks, manifold learning, semi-supervised learning

## Abstract

We propose a new clustering method based on a deep neural network. Given an unlabeled dataset and the number of clusters, our method directly groups the dataset into the given number of clusters in the original space. We use a conditional discrete probability distribution defined by a deep neural network as a statistical model. Our strategy is first to estimate the cluster labels of unlabeled data points selected from a high-density region, and then to conduct semi-supervised learning to train the model by using the estimated cluster labels and the remaining unlabeled data points. Lastly, by using the trained model, we obtain the estimated cluster labels of all given unlabeled data points. The advantage of our method is that it does not require key conditions. Existing clustering methods with deep neural networks assume that the cluster balance of a given dataset is uniform. Moreover, it also can be applied to various data domains as long as the data is expressed by a feature vector. In addition, it is observed that our method is robust against outliers. Therefore, the proposed method is expected to perform, on average, better than previous methods. We conducted numerical experiments on five commonly used datasets to confirm the effectiveness of the proposed method.

## 1. Introduction

Clustering is one of the oldest machine-learning fields, where the objective is, given data points, to group them into clusters according to some measure. Many clustering methods have been proposed for a long while [1], and been applied to real-world problems [2].

The best known classical methods are k-means [3] and Gaussian Mixture Model (GMM) clustering [4]. Though those methods are computationally efficient, they can only model convex shapes and are thus applicable in limited cases. The kernel k-means [5], kernel GMM clustering [6] and Spectral Clustering (SC) [7] can capture more complicated shapes than k-means and GMM but are difficult to scale up to large datasets. In recent years, due to technological progress, we can acquire many types of data such as images, texts, and genomes in large numbers. Thus, the demand of advanced efficient clustering methods grows even stronger [8].

Thanks to the development of deep neural networks, we can now handle large datasets with complicated shapes [9]. Consequently, the studies of clustering using deep neural networks has been proposed. One major direction in the studies is to combine deep AutoEncoders (AE) [10] with classical clustering methods. This AE is used to obtain a clustering friendly low dimensional representation. Another major direction is directly grouping a given unlabeled dataset into the clusters in the original input space by employing a deep neural network to model the distribution of cluster labels.

With both directions, there exist popular methods. We summarize their applicable data domain and well performing condition in Table 1. For examples, CatGAN (Categorical Generative Adversarial Networks) learns discriminative neural network classifiers that maximize mutual information between the input data points and the cluster labels, while enforcing the robustness of the classifiers to data points produced by adversarial generative models. Since maximizing mutual information implicitly encourages the cluster-balance distribution of the model to be uniform, CatGAN performs well under the condition of the uniform cluster balance. JULE (Joint Unsupervised LEarning) learns a clustering friendly low dimensional representation for image datasets by using a convolutional neural network [11]. The assigned cluster labels and low dimensional representation are jointly optimized by updating a n×n similarity matrix of the representations, where *n* is the number of data points. Thus, $O(n^{2})$ memory space must be secured to conduct the method.

As we can see in Table 1, most of their key conditions are not always realistic since the details of given unlabeled datasets are unknown and their size is large in typical machine-learning scenarios. On the other hand, SpectralNet does not require key condition. It only requires the following two fundamental assumptions: the smoothness and manifold assumptions [18]. Please note that the other methods in Table 1 also require the two assumptions. As for the weakness of SpectralNet, it is not robust against outliers. In the learning process, it learns the pairwise similarities over all data points. Therefore, the existence of outliers disturbs the method learning the similarities precisely, and thus returns inaccurate clustering results.

In this paper, we propose a deep clustering method named *Spectral Embedded Deep Clustering* (SEDC). Given an unlabeled dataset and the number of clusters, SEDC directly groups the dataset into the given number clusters in the input space. Our statistical model is the conditional discrete probability distribution, which is defined by a fully connected deep neural network. SEDC does not require key condition except the smoothness and manifold assumptions, and it can be applied to various data domains. Moreover, throughout our numerical experiments, we observed that our method was more robust against outliers than SpectralNet.

The procedure of SEDC is composed of two stages. In the first stage, we conduct SC only on the unlabeled data points selected from high-density region by using the geodesic metric to estimate the cluster labels. This special type of SC is named as *Selective Geodesic Spectral Clustering* (SGSC), which we propose for assisting SEDC as well. Thereafter, we conduct semi-supervised learning to train the model by using the estimated cluster labels and the remaining unlabeled data points. Please note that in this semi-supervised learning, we treat the estimated cluster labels of the selected unlabeled data points as the given true cluster labels. At last, by using the trained model, we obtain the estimated cluster labels of all given unlabeled data points.

In the remainder of this paper, we introduce related works in Section 2. We then introduce our proposed method in Section 3. We demonstrate the efficiency of our method with numerical experiments in Section 4. Finally, in Section 5, we conclude the paper with the discussion on future works.

## 2. Related Works

In this section, we first introduce the existing clustering studies based on deep neural networks. As mentioned in Section 1, there are two major directions in recent studies, i.e., the deep-AE-based clustering and the direct deep clustering. We then introduce the two techniques related to our proposed method, i.e., SC and Virtual Adversarial Training (VAT).

### 2.1. Existing Clustering Methods Using Deep Neural Network

In deep-AE-based clustering, the AE and a classical clustering method such as k-means are combined. The combination strategies are either sequential [19,20] or simultaneous [12,13,14]. In the sequential way, deeply embedded representations of the given dataset are obtained by the deep AE, and then a classical clustering method is applied to the embedded set. In the simultaneous way, the deep representations and their cluster labels are learned jointly by optimizing a single objective function.

As examples of the simultaneous way, we introduce [12,14] here. The method of [12] is named Deep Embedded Clustering (DEC). DEC trains a deep neural network by iteratively minimizing the Kullback–Leibler (KL) divergence between a centroid-based probability distribution and an auxiliary target distribution. The deep network is used as the AE. They reported the clustering performance of DEC depended on the initial embedded representations and cluster centroids obtained by the AE and k-means. The method of [14] is named Variational Deep Embedding (VaDE). The approach relies on a Variational AutoEncoder (VAE) [21] that uses a Gaussian mixture prior. VaDE trains its deep neural network by minimizing the reconstruction error, while enforcing that the low dimensional representations follow the Gaussian mixture model.

Regarding the direct deep clustering, we introduce [15,17]. The method of [15] is named Information Maximizing Self-Augmented Training (IMSAT). It is based on data augmentation, where a deep neural network is trained to maximize the mutual information while regularizing the network so that the cluster label assignment of original data will be consistent with the assignment of augmented data. The method of [17] is named SpectralNet. This method is proposed to overcome the scalability and generalization of SC. It uses two deep neural networks. The first network learns the similarities among all given data points. This network is known as Siamese net [22]. Then, the second network learns a dimension reduction mapping which preserves the similarities obtained by the first net. After both are trained, the dimensionality reduced data points obtained by the second network are grouped into clusters by k-means.

### 2.2. Related Techniques with Proposed Method

SEDC handles the following clustering problem: given a set of unlabeled data points $X={\left\{x_{i}\right\}}_{i=1}^{n}$ ($x_{i}\in R^{D}$) and the number of clusters *C*, group X into *C* clusters. In SEDC, the estimated cluster label of $x_{i}$ is obtained by the trained conditional discrete probability distributional classifier. This classifier is denoted by $p_{\theta }\left(y|x\right)$ where θ, $x_{i}$ and *y* are a set of parameters, a feature vector and a cluster label, respectively. The cluster label *y* ranges {1,⋯,C}. In addition, the classifier is defined by a fully connected deep neural network whose last layer is the soft-max function. SC and VAT, explained below, take an important role in SEDC: see the detail in Section 3.

#### 2.2.1. Spectral Clustering

SC [7,23] is a popular classical clustering algorithm. We here introduce the commonly used framework of SC, which is used also in SEDC algorithm. It first embeds the data points in the eigenspace of the Laplacian matrix derived from the pairwise similarities over all given data points. Then, SC applies k-means on the representation to obtain the cluster labels. The SC algorithm is outlined below.

Given dataset X, define the weighted undirected graph *G* which comprises a set of vertices X and a set of undirected edges *E* defined by $k_{1}$-nearest neighbor ($k_{1}$-NN) based on a metric *d*. Suppose that each edge $e_{ij}\in E$ has a non-negative symmetric weight $w_{ij}$.
Denote the n×n affinity matrix $D^{-1/2}WD^{-1/2}$ on *G* by L, where $W={\left(w_{ij}\right)}_{i,j}$, and D is the diagonal matrix whose entries are given by $d_{ii}=\sum_{j}w_{ij}$.
Given the number of clusters *C*, compute the largest *C* eigenvectors $u_{1},\cdots ,u_{C}$ of the eigenproblem Lu=λu. Let $U\in R^{n\times C}$ be the matrix containing the vectors $u_{1},\cdots ,u_{C}$ as columns. Thereafter, re-normalize each row of U to have unit length.
(4) Cluster *n* rows of U as points in $R^{C}$ by conducting k-means (k =C). Let ${\left\{{\hat{y}}_{i}\right\}}_{i=1}^{n}$ and ${\left\{\mu_{j}\right\}}_{j=1}^{C}$ be the estimated labels of X and the set of centroids obtained by k-means, respectively.

The above procedure can be noted as $\left({\left\{{\hat{y}}_{i}\right\}}_{i=1}^{n},{\left\{\mu_{j}\right\}}_{j=1}^{C},U\right)=\mathrm{SC}\left(X,k_{1},d,w,C\right)$. The weight *w* is often defined by the following similarity function:

(1) $$w_{ij}=\left\{\begin{array}{cc} \exp(-d{\left(x_{i},x_{j}\right)}^{2}/\sigma^{2}), & \;e_{ij}\in E,\\ 0, & \;e_{ij}\notin E, \end{array}\right.$$

The bandwidth σ is selected by the median or mean heuristics [24]. In our proposed method, we employ k-means++ [25] technique since the method uses that SC function.

#### 2.2.2. Virtual Adversarial Training

VAT [26] is a regularization method based on local perturbation. It forces the statistical model $p_{\theta }\left(y|x\right)$ follow the smoothness assumption. VAT is known to empirically perform better than other local perturbation methods such as random perturbation [27] and adversarial training [28] in both semi-supervised and supervised learning scenarios. It can be employed also in unsupervised learning scenarios [15,29] since VAT only requires the unlabeled data points.

VAT first defines the adversarial point $T_{\mathrm{VAT}}\left(x\right)$ with given x as follows:

(2) $$T_{\mathrm{VAT}}\left(x\right)=x+r_{\mathrm{vadv}},$$

where $r_{\mathrm{vadv}}$ is ϵ-perturbation to a virtual adversarial direction:

(3) $$r_{\mathrm{vadv}}=\underset{r}{\mathrm{argmax}}\left\{\mathrm{KL}\left[p_{\theta_{t}}\left(y|x\right)∥p_{\theta }\left(y|x+r\right)\right];{∥r∥}_{2}\leq \epsilon \right\}.$$

In Equation (3), $\theta_{t}$ is the estimated parameter at *t*-th iteration, and KL is Kullback–Leibler divergence [30]. Then, VAT minimizes the following $R_{\mathrm{VAT}}\left(\theta ;X\right)$ with respect to θ:

(4) $$R_{\mathrm{VAT}}\left(\theta ;X\right)=\frac{1}{n}\sum_{i=1}^{n}\mathrm{KL}\left[p_{\theta_{t}}\left(y|x_{i}\right)∥p_{\theta }\left(y|T_{\mathrm{VAT}}\left(x_{i}\right)\right)\right].$$

The approximation of $r_{\mathrm{vadv}}$ in Equation (3) is computed by the following two steps:

(5) $$\begin{array}{ccc} g & \leftarrow & {\left.\nabla_{r}\;\mathrm{KL}\left[p_{\theta_{t}}\left(y|x\right))∥p_{\theta_{t}}\left(y|x+r\right)\right]\right|}_{r=\xi d}, \end{array}$$

(6) $$\begin{array}{ccc} r_{\;\mathrm{vadv}\;} & \approx & \epsilon \frac{g}{{∥g∥}_{2}}, \end{array}$$

where $d\in R^{D}$ is a random unit vector generated by the standard normal distribution, and $\xi \in R_{+}$ is a fixed small positive number. Regarding the logic behind the approximation, see Section 3.3 of [26].

**Remark** **1.** *The radius ϵ of Equation (*3*) is defined for given x as below:*

(7) $$\epsilon \left(x\right)=\alpha ∥x-x^{\left(z\right)}{∥}_{2},$$

*where α is a scalar and $x^{\left(z\right)}$ is the z-th nearest data point from x. In [15], α=0.4 and z=10 are used.*

## 3. Proposed Method

As we already mentioned in the end of Section 1 and the beginning of Section 2.2, given an unlabeled dataset $X={\left\{x_{i}\right\}}_{i=1}^{n}$ ($x_{i}\in R^{D}$) and the number of clusters *C*, our proposed deep clustering named SEDC groups X into *C* clusters. Since this grouping is achieved by obtaining the estimated cluster labels of X, our goal can be replaced by estimating the cluster labels up to permutation of labels. In SEDC, the estimated cluster label of each $x_{i}\in X$ is defined by ${\mathrm{argmax}}_{j=1,\ldots ,C}p_{\theta^{*}}\left(y=j|x_{i}\right)$, where $\theta^{*}$ is the trained set of parameters. The training scheme of the classifier $p_{\theta }\left(y|x\right)$ is as follows: we firstly only estimate the cluster labels of selected unlabeled data points by using only X (this part is done by SGSC algorithm.), and then conduct semi-supervised learning to train the classifier. Regarding with this semi-supervised learning, we use the estimated cluster labels of selected unlabeled data points and the remaining unlabeled data points, which are treated as the given true cluster labels and unlabeled data points respectively.

In this section, we first introduce SGSC. Thereafter, we present our main method SEDC.

### 3.1. Selective Geodesic Spectral Clustering

Using SGSC, the clustering problem is converted into a semi-supervised learning problem as shown below. In SGSC, firstly some unlabeled data points are selected from high-density regions in the original dataset. Then, the SC is used to the selected unlabeled data points with the geodesic metric. As a result, we obtain cluster labels on the selected points. Since the points are picked up from the high-density regions, the locations of selected points are stable and robust against outliers [31]. The geodesic metric is approximated by the graph shortest path distances on the graph. The reason to employ the geodesic metric is that the metric is known to be useful to capture the structure of the data manifolds especially when the number of given data points is large [32]. Here, the number of selected data points is a hyperparameter in SGSC. Suppose that the hyperparameters are tuned appropriately, Then, the set of the selected data points with their estimated cluster labels can roughly approximate the manifolds represented by the full original dataset even when the dataset has complicated manifolds inluding outliers.

Throughout numerical experiments on five datasets in later section, the following two are confirmed. Firstly, the estimation accuracy of cluster labels with selected points by SGSC can stay high. Then, secondly, due to the highly accurate estimation by SGSC, it can help the clustering by SEDC to be successful on several types of datasets on average.

We will refer to the selected data points as *hub* data points. Let H⊂X be the set of hub data points. The hub dataset *H* is formally defined below.

**Definition** **1.** *Let X be the given unlabeled dataset. On the graph G=(X,E), let $N_{j}$ be the set of adjacent nodes of $x_{j}\in X$. For a natural number h, the hub set H is defined as the collection of nodes that ranked in the top-h cardinality of $N_{j}$ in X.*

**Figure 1:**
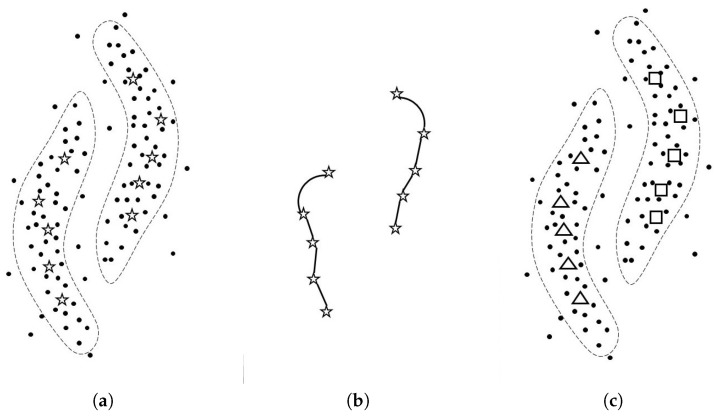
This figure explains how SGSC works. (**a**): Given unlabeled data points, in line 2 of Algorithm 1, SGSC computes the hub data points. The hub data points are expressed by star symbols, and the number of hub data points *h* is ten in this case; (**b**): In line 4 of the algorithm, SGSC focuses only on the hub data points, then conducts SC with the geodesic metric on those hub points, where we set one to $k_{1}$; (**c**): As the results, we obtain the cluster labels of hub points. The triangle and square symbols mean different labels. Please note that an actual output of SGSC is the estimated conditional discrete probability distributions with hub data points, but we can obtain the estimated cluster labels from the distributions.

Algorithm 1 and Figure 1 show the pseudo code of SGSC and the mechanism of SGSC, respectively. The detail of this algorithm is explained below.


**Algorithm 1:**
$Q=\mathrm{SGSC}(X,k_{0},k_{1},h,C)$

 **Input**: Unlabeled dataset $X={\left\{x_{i}\right\}}_{i=1}^{n}$. Number of neighbors $k_{0},k_{1}$. Number of hub data points *h*. Number of clusters *C*. **Output**: The estimated conditional discrete probability distributions with hub data points, Q.
1:Construct the undirected graph $G_{0}=\left(X,E\right)$, where the edge set *E* is defined by $k_{0}$-NN with the Euclidean distance.2:Build the hub dataset *H* on graph $G_{0}$ such that |H|=h. Denote the element of *H* by $x_{\left(i\right)}$ (i=1,⋯,h).3:Define the geodesic metric $d_{G_{0}}$ as the shortest path distance on the graph $G_{0}$.4:Define ${\left\{\mu_{j}\right\}}_{j=1}^{C}$ and U as the two outputs of $\mathrm{SC}(H,k_{1},d_{G_{0}},w,C)$, where the weight $w(x_{i},x_{j})$ is defined by $\exp(-d_{G_{0}}{\left(x_{i},x_{j}\right)}^{2}/\sigma^{2})$. Then, compute the conditional cluster probability $q_{j|(i)}$ with each hub data point $x_{\left(i\right)}$ in *H* as follows:
$$q_{j|(i)}=\frac{{\left(1+{∥{\tilde{x}}_{\left(i\right)}-\mu_{j}∥}_{2}^{2}/\gamma \right)}^{-\frac{\gamma +1}{2}}}{\sum_{j^{\prime }=1}^{C}{\left(1+{∥{\tilde{x}}_{\left(i\right)}-\mu_{j^{\prime }}∥}_{2}^{2}/\gamma \right)}^{-\frac{\gamma +1}{2}}},$$
where γ is a small positive number and ${\tilde{x}}_{\left(i\right)}$ is *i*-th row of U.5:Let $q_{\left(i\right)}$ and Q be $\left(q_{1|(i)},\cdots ,q_{C|(i)}\right)$ and h×C matrix, respectively. The *i*-th row of Q is defined by $q_{\left(i\right)}$.

- Line 1: Given an unlabeled dataset X, the undirected graph $G_{0}$ is constructed in the $k_{0}$-nearest neighbor ($k_{0}$-NN) manner with the Euclidean metric. $G_{0}$ is used not only for defining the hub set *H* but also for approximating the geodesic distance on the manifolds shaped by X. We consider $k_{0}$ as a hyperparameter.
- Line 2: Given the number of hub points *h* and $G_{0}$, the algorithm defines the hub set *H* based on Definition 1. Outliers can be excluded from *H* by setting *h* appropriately. In this algorithm, *h* is considered to be a hyperparameter.
- Line 3: The geodesic distance is determined from the Euclidean distances on the undirected edges of $G_{0}$. In Line 4, we need to compute the geodesic distances between the data points of *H*. Efficient algorithms are available for this purpose [32,33].
- Line 4: Given the number of clusters *C*, we estimate the conditional discrete probability distribution $p(y|x_{\left(i\right)})$ for each $x_{\left(i\right)}\in H$, where *y* is the cluster label ranging {1,⋯,C}. The estimated $p(y|x_{\left(i\right)})$ is denoted as $q_{\left(i\right)}=\left(q_{1|(i)},\cdots ,q_{C|(i)}\right)$. This estimation relies on conducting SC with $d_{G_{0}}$ metric only on *H*. The definition of the weight *w* in this SC follows Equation (1). The key to succeed the estimation is to employ the combination of a different number of neighbors $k_{1}$ from $k_{0}$ and the geodesic metric $d_{G_{0}}$ to a SC. Typically, given data points that are dense in the input space, the combination of a small number of neighbors and the Euclidean metric makes a SC perform well. However, we consider *H*, which is sparse in the input space. Therefore, we employ the combination. We consider $k_{1}$ as a hyperparameter as well. Following [34], we compute $q_{j|(i)}$ by using the outputs ${\left\{\mu_{j}\right\}}_{j=1}^{C}$ and U of $\mathrm{SC}(H,k_{1},d_{G_{0}},w,C)$. Please note that $q_{j|(i)}$ can be considered to be the probability that ${\tilde{x}}_{i}$ belongs to the cluster *j*, where ${\tilde{x}}_{\left(i\right)}$ is the low dimensional representation of $x_{\left(i\right)}$ according to the property of SC [23]. As for γ, we set $10^{-60}$ to it.

**Remark** **2.** *Though we say we estimate the “cluster labels” of hub data points by SGSC, it actually outputs the estimated conditional probability distributions with hub data points. The reason is that throughout our preliminary experiments, we observed that employing Q of line 5 made SEDC perform better than employing the one-hot vector. This one-hot vector, for instance, can be defined by using the estimated cluster labels ${\left\{{\hat{y}}_{\left(i\right)}\right\}}_{i=1}^{h}$ which is one of the outputs of $\mathrm{SC}(H,k_{1},d_{G_{0}},w,C)$.*

### 3.2. Spectral Embedded Deep Clustering

SEDC is a deep clustering method for clustering. Given an unlabeled dataset $X={\left\{x_{i}\right\}}_{i=1}^{n}$ and the number of clusters *C*, it groups X into *C* clusters. As mentioned in the beginning of this section, this method employs the conditional discrete probability distribution $p_{\theta }\left(y|x\right)$ as the statistical model, which is defined by a fully connected deep neural network. By using the trained model, we obtain the estimated cluster label of each $x_{i}$. This method does not require an additional condition except two fundamental assumptions: the smoothness and manifold assumptions. Therefore, among the methods of Table 1, only SpectralNet is comparable to SEDC in this point. In addition, our method can be applied to various data domains once the raw data is transformed to the feature vector. Furthermore, empirically speaking, the performance of SEDC can be robust against outliers due to the robustness of SGSC against them. The pseudo code of SEDC is shown in Algorithm 2. The explanation is below.

The procedure of SEDC is composed of two stages. In the first stage, we estimate the conditional discrete probability distributions Q with hub data points. In the second stage, by treating Q as the given true distributions of hub data points, we conduct semi-supervised learning where Q and the remaining unlabeled data points are used, to train the statistical model $p_{\theta }\left(y|x\right)$. After this training, SEDC returns the estimated cluster labels of each $x_{i}\in X$ by ${\mathrm{argmax}}_{j=1,\ldots ,C}p_{\theta^{*}}\left(y=j|x_{i}\right)$, where $\theta^{*}$ is the trained set of parameters and j∈{1,⋯,C}. The estimated cluster labels of $x_{i}$ is denoted by ${\hat{y}}_{i}$. Note that the estimated cluster labels of hub data points might be updated at the end of SEDC procedure.

In the second stage, we conduct semi-supervised learning to train the statistical model $p_{\theta }\left(y|x\right)$ using $q_{\left(i\right)},i=1,\ldots ,h$. Recall that the model $p_{\theta }\left(y|x\right)$ is defined by the deep neural network whose last layer is soft-max function. The number of neurons of the first and last layer are the dimension of feature vector *D* and the number of clusters *C*, respectively. In this semi-supervised learning, we minimize the following loss with respect to θ:

(8) $$R_{\mathrm{VAT}}\left(\theta ;X\right)+\frac{\lambda_{1}}{h}\sum_{i=1}^{h}\mathrm{KL}\left[p_{\theta }\left(y|x_{\left(i\right)}\right)∥q_{\left(i\right)}\right]+\lambda_{2}H\left(Y|X\right),$$

where $\lambda_{1}$ and $\lambda_{2}$ are hyperparameters that range over positive numbers. In Equation (8), the first and second terms express VAT loss of Equation (4) and the pseudo empirical loss with estimated cluster probabilities of hub data points, respectively. The third term is the conditional Shannon entropy [30] averaged over X, and it is defined as follows:

$$H\left(Y|X\right)=-\frac{1}{n}\sum_{i=1}^{n}\sum_{j=1}^{C}p_{\theta }\left(y=j|x_{i}\right)\log p_{\theta }\left(y=j|x_{i}\right).$$

**Algorithm 2:**
${\left\{{\hat{y}}_{i}\right\}}_{i=1}^{n}=\mathrm{SEDC}\left(X,k_{0},k_{1},h,C,\lambda_{1},\lambda_{2}\right)$

 **Input**: Unlabeled dataset $X={\left\{x_{i}\right\}}_{i=1}^{n}$. Number of neighbors $k_{0},k_{1}$. Number of hub data points *h*. Number of clusters *C*. Regularization parameters $\lambda_{1},\lambda_{2}>0$. **Output**: The estimated cluster labels of X, ${\left\{{\hat{y}}_{i}\right\}}_{i=1}^{n}$.
1:Obtain the h×C matrix of estimated conditional discrete probability distributions with hub data points Q by computing $\mathrm{SGSC}(X,k_{0},k_{1},h,C)$ of Algorithm 1. Denote *i*-th row of Q by $q_{\left(i\right)}$, which means the estimated cluster label probability distribution of hub data point $x_{\left(i\right)}$. The index *i* ranges {1,⋯,h}.2:Let $p_{\theta }\left(y|x\right)$ be a statistical model, which is the cluster label probability distribution with given data point x. The cluster label ranges {1,⋯,C}. Define the objective of Equation (8) by using $p_{\theta }\left(y|x\right)$, ${\left\{q_{\left(i\right)}\right\}}_{i=1}^{h}$ and given $\lambda_{1},\lambda_{2}$. Then, minimize the objective with θ in stochastic gradient descent fashion. Denote the optimized parameter by $\theta^{*}$.3:Obtain the estimated cluster labels of all data points in X by using the trained classifier $p_{\theta^{*}}\left(y|x\right)$. Denote $p_{\theta^{*}}\left(y=j|x_{i}\right)$ by $p_{j|i}^{*}$. Then, for all data point index *i*, compute ${\hat{y}}_{i}$ by ${\hat{y}}_{i}=\underset{j}{\mathrm{argmax}}\;p_{j|i}^{*}.$


We use the Adam optimizer [35] for the minimization. After minimizing Equation (8), we estimate the labels of X by using the trained parameter $\theta^{*}$. Let ${\left\{{\hat{y}}_{i}\right\}}_{i=1}^{n}$ denote the estimated cluster labels of $X={\left\{x_{i}\right\}}_{i=1}^{n}$. The labels are computed as follows: ${\hat{y}}_{i}={\mathrm{argmax}}_{j=1,\ldots ,C}\;p_{\theta^{*}}\left(y=j|x_{i}\right)$.

As mentioned in Section 2.2, the minimization of the VAT loss encourages $p_{\theta }\left(y|x\right)$ to follow the smoothness assumption. In addition, that of entropy loss helps the model to follow the cluster assumption [18]. The cluster assumption says that true decision boundary is not located in regions of the input space that are densely populated with data points. The entropy loss is commonly used in many studies [15,26,36,37]. Please note that the entropy loss is defined only by using the unlabeled data points, like VAT loss. With regard to the pseudo empirical loss, we can consider other candidates such as the cross entropy. The reason we chose the KL-divergence is that we observed that the KL-divergence made SEDC perform better than other candidates in our preliminary experiments.

### 3.3. Computational and Space Complexity of SEDC

The procedure of SEDC is composed of two stages. The first stage (line 1 of Algorithm 2) is conducting SGSC algorithm. The second stage (line 2 of Algorithm 2) is training the model by optimizing Equation (8). Therefore, total computational complexity of SEDC is the summation of the total computational complexity of SGSC and the complexity consumed in the mini-batch training. In the following, we analyze the computational complexity consumed in each line of Algorithm 1: see this summary in Table 2. Suppose that $h,k_{0},k_{1}\ll n$. In line 1 of the algorithm, we consume $O(Dn^{2})$ to construct $k_{0}$-NN graph [38], where *D* is the dimension of feature vector. Then, in the line 2, we consume O(nlogn) because we sort the nodes of the graph in descending order of degree for defining the hub set *H*. Then, in the line 3, we consume $O\left(h(\log n+k_{0})n\right)$ for computing the graph shortest path distances between the data points in *H*: see algorithm 1 of [32]. Thereafter, in the line 4, we consume $O(h^{3})$ for solving the eigenvector problem of the Laplacian matrix.

As for the memory complexity, since the dominant factors are to save $k_{0}$-NN graph and the model, SEDC needs $O\left(\max\{k_{0}n,|\theta |\}\right)$ where θ is the set of parameters in a deep neural network.

**Remark** **3.** *For most of deep clustering methods relying on k-NN graph construction, the dominant factor with their total computational complexity is k-NN graph construction, i.e., we need $O(Dn^{2})$. However, according to [39,40], by constructing the approximated k-NN graph, we only need O(Dnlogn) for the construction.*

## 4. Numerical Experiments

In this section, we show the results of our numerical experiments. First, we show how accurately SGSC can estimate the labels of hub data points on five datasets. Then, we show the clustering results on the same five datasets by SEDC. With regards to the clustering experiments, we compare our proposed method with five popular methods: k-means [3], SC [7], DEC [12], IMSAT [15] and SpectralNet [17].

### 4.1. Datasets and Evaluation Metric

We conducted experiments on five datasets. Two of them are real-world datasets named MNIST [41] and Reuters-10k [42]. The other three are synthetic datasets named FC, TM, and TR. A summary of the dataset statistics is described in Table 3.

**Figure 2:**
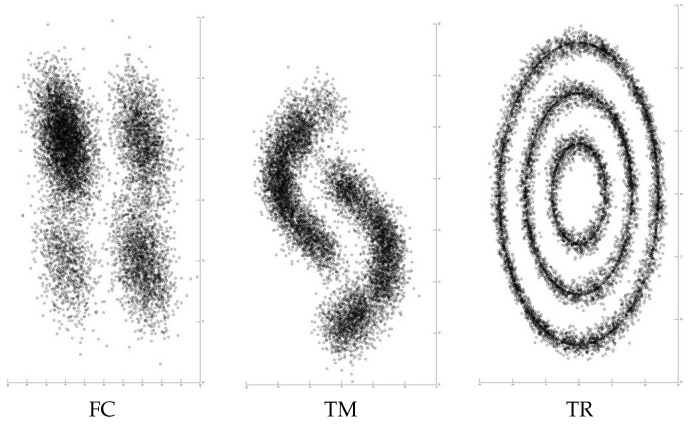
The generated examples of three commonly used synthetic datasets. The left, middle and right pictures correspond the examples of four clusters (FC), two moons (TM) and three rings (TR), respectively.

MNIST is a collection of 28 × 28 gray-scale images of handwritten digits. It is composed of 60,000 training and 10,000 test sets. The digits are transformed to 784-dimensional feature vectors. Then, they are centered, and the size is normalized. In this experiment, we use all 70,000 data points. Reuters-10k is a dataset of English news stories labeled with a category tree [42]. Following [12], we used the same four root labels. The news stories are transformed to feature vectors by computing the TF-IDF features on the 2000 most frequent words. This dataset contains 685,071 documents. In this experiment, random subsets of 10,000 samples from the full dataset are drawn. As for the synthetic datasets, we show the generated examples in Figure 2. An example of FC is the left picture in the figure. This dataset is composed of simple four clusters with some outliers. The cluster balance is biased. The ratio of biggest cluster is 50% to the dataset, then 20%,20% and 10%. The four clusters are close to each other. An example of TM is middle picture in the table. This dataset includes some outliers and its decision boundary is non-linear. The cluster balance is uniform. The example of TR is the right picture in Table Figure 2. This dataset is composed of three concentric rings. The ratio of outer ring to the whole size is 50%, and the middle and inner ones are 33% and 17%, respectively.

Regarding the evaluation metric, since we are in unsupervised learning scenario, we adopt the standard metric for evaluating clustering performance [12,15], which measures how close the estimated cluster labels are to the ground truth. For an unlabeled dataset ${\left\{x_{i}\right\}}_{i=1}^{n}$, let ${\left\{y_{i}\right\}}_{i=1}^{n}$ and ${\left\{{\hat{y}}_{i}\right\}}_{i=1}^{n}$ be its true cluster label set and estimated cluster label set, respectively. The number of data points is denoted by *n*. Suppose that the both true and estimated cluster labels $y_{i}$ and ${\hat{y}}_{i}$ take the same range, i.e., {1,2,⋯,C}. The clustering accuracy (ACC) is then defined by

(9) $$\mathrm{ACC}=\max_{\tau }\frac{\sum_{i=1}^{n}1\left[y_{i}=\tau \left({\hat{y}}_{i}\right)\right]}{n},$$

where τ ranges over all permutations of cluster labels, and 1[⋯] is the indicator function given by

(10) $$1\left[y_{i}=\tau \left({\hat{y}}_{i}\right)\right]=\left\{\begin{array}{cc} 1, & \;y_{i}=\tau \left({\hat{y}}_{i}\right),\\ 0, & \;y_{i}\neq \tau \left({\hat{y}}_{i}\right). \end{array}\right.$$

The optimal assignment of τ can be computed using the Kuhn–Munkres algorithm [43].

### 4.2. Performance Evaluation of SGSC

We here show how accurately SGSC could estimate the cluster labels of five datasets in Table 3. Strictly speaking, SGSC of Algorithm 1 has five hyperparameters, which are $k_{0}$, σ of Equation (1), $k_{1}$, *h* and γ. In this experiment, we nevertheless do not consider σ and γ as the hyperparameters. The reason is, when median heuristics and $10^{-60}$ were employed to σ and γ respectively, SGSC performed well throughout all datasets of Table 3 in our preliminary experiments. Thus, we consider the rest three as the hyperparameters.

Generally speaking, in unsupervised learning, it is difficult to tune hyperparameters because we cannot conduct cross-validation. However, by using the idea of transfer learning, we can ease the difficulty. Following [15], we tune the three aforementioned hyperparameters. Let Λ be the triplet $\left(k_{0},k_{1},h\right)$. The best one is denoted by $\Lambda^{*}=\left(k_{0}^{*},k_{1}^{*},h^{*}\right)$. Now, $\Lambda^{*}$ is defined as follows:

(11) $$\Lambda^{*}=\underset{\Lambda }{\mathrm{argmax}}\sum_{i}\frac{\mathrm{ACC}(\Lambda ,{\mathrm{dataset}}_{i})}{\mathrm{ACC}(\Lambda_{{\mathrm{dataset}}_{i}}^{*},{\mathrm{dataset}}_{i})},$$

where ${\mathrm{dataset}}_{i}$ is *i*-th source domain. $\Lambda_{{\mathrm{dataset}}_{i}}^{*}$ is the best hyperparameter for the ${\mathrm{dataset}}_{i}$. $\mathrm{ACC}(\Lambda ,{\mathrm{dataset}}_{i})$ is the clustering accuracy of Equation (9) when the hyperparameter Λ is selected for ${\mathrm{dataset}}_{i}$. The dataset on which we want to compute the clusters will be referred to as target domain. The source domain of each given dataset is shown in Table 4. USPS [44] is a dataset of handwritten digit. The number of clusters is ten. 20news [15] is a dataset of newsgroup documents, which is partitioned nearly evenly across 20 different newsgroups. The source domain of FC is FC, TM, and TR. Please note that FC, TM, and TR used as sources have slightly different generating rules compared to target ones. The same things goes for the target TM and TR datasets. By using these source domains, we tune the hyperparameters.

As for the ranges of candidates with three hyperparameters, we define $k_{0}\in \left\{10\times i_{0}\;|\;i_{0}=1,\cdots ,10\right\}$, $k_{1}\in \left\{5\times i_{1}\;|\;i_{1}=1,\cdots ,5\right\}$ and $h\in \{100\times i_{2}\;|\;i_{2}=2,\cdots ,5\}$. By conducting tuning technique of Equation (11) on each source domain, we obtained the best hyperparameters for given datasets of Table 3 as follows: The best ones for MNIST, Reuters-10k, FC, TM, and TR are $\left(k_{0}^{*},k_{1}^{*},h^{*}\right)=\left(10,10,500\right)$, (100,20,200), (10,10,500), (10,10,500) and (10,10,500) respectively. For an example, when $k_{0}=10$, we obtained the accuracy matrix for both MNIST and USPS shown in Table 5. According to this table, the best pair $\left(k_{1}^{*},h^{*}\right)=\left(10,500\right)$ of USPS is transferred to MNIST. Finally, based on the above best pair of hyperparameters, we conduct SGSC on each target dataset. The results are shown in Table 6.

### 4.3. Performance Evaluation of SEDC

We here show how accurately SEDC can cluster the five given datasets of Table 3, and how to proceed in implementing it. Across all the datasets, we define the network structure of $p_{\theta }\left(y|x\right)$ by *D*-1200-1200-*C*, where *D* and *C* are the dimension of feature vector and the number of clusters, respectively. We used ReLU [45] for all the activation functions, and employed batch normalization technique [46] on each layer. For the Adam optimizer, we set the learning rate to 0.002. Following [47], we initialized the bias term and the weights of directed edges in the deep neural network as follows: each weight is initialized by the value of a Gaussian distribution with a mean of 0, and standard deviation of $\delta \times \sqrt{2/f_{\mathrm{in}}}$, where $f_{\mathrm{in}}$ is the number of input neurons. We set the δ to $10^{-1}$-$10^{-1}$-$10^{-4}$ for weight matrices from the input to the output. The all bias terms were initialized to 0. The number of epochs is fixed to 25, and each epoch is made by 100 iterations. As for the mini-batch with Equation (8), in each iteration, we sample h/10 and (n-h)/100 data points from the pseudo labeled dataset and the unlabeled dataset, respectively. The pseudo labeled samples are used for approximating the second term of Equation (8), and both pseudo labeled and unlabeled samples are used for approximating the first and third terms of Equation (8). Moreover, in VAT, we set ξ to ten in Equation (5). The radius ϵ is defined by the same way as [15]: see Remark 1.

With respect to the selection of hyperparameters in SEDC, since we have already finished to tune $k_{0},k_{1}$ and *h* in the previous section, we only focus on the remaining hyperparameters $\lambda_{1}$ and $\lambda_{2}$. The tuning tactic is also based on Equation (11), and the source domains of Table 4 are used for the tuning. The ranges of candidates are defined by as follows: $\lambda_{1}\in \left\{0.1\times j_{1}\;|\;j_{1}=1,\cdots ,10\right\}$, $\lambda_{2}\in \left\{0.1\times j_{2}\;|\;j_{1}=1,\cdots ,10\right\}$. After the tuning, we obtained $\lambda_{1}=\lambda_{2}=1$ as the best ones for all datasets. By using the tuned hyperparameters and computing the SEDC, we get the results shown in Table 7.

As we can see in Table 7, our method averagely performs better than other methods. One of the reason is that we do not require key conditions to SEDC. For an example, IMSAT does not perform well for datasets with non-uniform cluster balance such as TR. In addition to the wide applicability, another reason lies on the robustness against outliers. When we see the performance of SEDC on FC which includes outliers, the method is more robust against outliers compared to SpectralNet. In fact, SpectralNet suffered from the two datasets which include outliers. Please note that the hyperparameter tuning of SEDC hugely contributes to the robustness. On the other hand, if we see some columns of Table 7 such as MNIST and Reuters-10k, SEDC does not outperform IMSAT. The clustering accuracy of IMSAT with MNIST is known to be one of the best results among several deep clustering methods [48]. In addition to VAT regularization, the almost perfect prior knowledge of cluster balance with MNIST seems to greatly help IMSAT achieve such the result. Though our method employs similar objective function with IMSAT, since we use the estimated cluster labels, the estimation error degraded the performance of SEDC. As for Reuters-10k, we can list two reasons why SEDC does not outperform IMSAT well. The first one is that the number of given data points is not enough since the geodesic metric is approximated by the graph shortest path distance in SEDC. In fact, we observed that by using 20,000 unlabeled data points with Reuters, the clustering accuracy of SEDC and IMSAT were 0.739 (0.06) and 0.696 (0.05), respectively. The accuracy was the average of seven times experiments. The second one is that the source domain of Reuters-10k might be not appropriate since the cluster balances of 20new and Reuters are uniform and non-uniform, respectively.

In Table 8, we show the corresponding mean runtime and standard deviation of Table 7. As we can expect, k-means is the fastest clustering method among the six. The runtime of SC is quite long especially when the size of dataset is large. The most heavy computation is in solving the eigenvalue problem. IMSAT is the second slowest method in the six. Since this method require the *k*-NN graph for defining the adaptive radius with VAT: see Remark 1, this computation is dominant in the whole procedure. In addition to the time of graph construction, relatively larger number of epochs (50 epochs) for training the deep neural network also affected the total runtime. DEC is the fastest method among the deep clustering methods. After the pre-training, DEC simply trains the deep neural network by using mini-batches until the convergence. Please note that the shown runtimes of DEC do not include the runtimes consumed on the pre-training. If we combine the runtime of that pre-training with shown ones, the total runtimes of DEC will be much longer: see Section 4.3 of [12]. SpectralNet also computes *k*-NN graph. Therefore, the dominant part of computation is the graph construction. In addition to this constructing time, since the number of epochs for training the two deep neural networks are not large, the total runtime of SpectralNet is relatively fast among the four deep clustering methods. Regarding with SEDC, as we already mentioned in Section 3.3, the dominant part is computing *k*-NN graph. In addition to this computing, since we set 25 epochs to the training, the total runtimes is medium.

## 5. Conclusions and Future Work

In this paper, we propose a deep clustering method named SEDC. Given an unlabeled dataset and the number of clusters, the method groups the dataset into the given number clusters. Regarding its advantages, it does not require an additional condition except two fundamental assumptions: smoothness and manifolds assumptions. In this point, only SpectralNet of Table 1 is comparable. In addition, SEDC also can be applied to various data domains since it does not have preferred data domains, as long as raw data is transformed to feature vectors. Furthermore, the performance of SEDC can be robust against existence of outliers unlike SpectralNet. According to these advantages, our proposed method can be expected to averagely perform better than previous deep clustering methods. As a result, this expectation is empirically confirmed by conducting numerical experiments on five commonly used datasets: see Table 7. Therefore, we think our method can be a competitive candidate for users in some practical clustering scenarios where prior knowledge of the given unlabeled dataset is limited.

However, there are two main drawbacks. On the one hand, since the method needs hyperparameter tuning, if we do not have appropriate labeled source domains to learn them from and transfer, then it may fail. On the other hand, since the method requires the number of clusters, it does not work for datasets where nothing is known on the number of clusters such as genome datasets.

Finally, we discuss about our two future works. The first one is to invent a more noise-robust semi-supervised learning framework and then apply it to SEDC instead of Equation (8). Since some of the estimated cluster labels by SGSC are not perfectly accurate, we need to invent such the framework to stabilize the performance of SEDC. The second one is to modify our method for handling structured data, i.e., graph data or sequential data.

## Figures and Tables

**Table 1:** Summary of popular deep clustering methods. Three AE-based methods and four direct methods are presented, including our method SEDC. A method has a preferred domain when it is specialized for a certain type of data. Please note that all methods require the smoothness and manifold assumptions. Some methods require additional key conditions except the above two assumptions. Empty spaces mean “None”. All methods below require the number of clusters as one of their inputs.

AE-Based Methods	Preferred Domain	Key Condition
DEC [12]		AE acquires good representation.
JULE [13]	Image	Size of dataset is not large.
VaDE [14]		The representation follows GMM.
**Direct Methods**	**Preferred Domain**	**Key Condition**
IMSAT [15]		Cluster balance is uniform.
CatGAN [16]		Cluster balance is uniform.
SpectralNet [17]		
SEDC		

**Table 2:** The computational complexity of each line of Algorithm 1 (SGSC algorithm), where D,n,h and $k_{0}$ are the dimension of the feature vector, the number of given unlabeled data points, the number of hub data points and the number of neighbors, respectively.

Corresponding Line of Algorithm 1	Line 1	Line 2	Line 3	Line 4
Theoretical computational complexity	$O(Dn^{2})$	O(nlogn)	$O\left(h(\log n+k_{0})n\right)$	$O(h^{3})$

**Table 3:** Summary of dataset statistics. #Points is the number of data points used for the training of each clustering method. #Clusters is the given number of clusters. Dimension is the dimension of given feature vector. %Largest cluster means the percentage of the largest cluster size to each size of dataset.

Dataset	#Points	#Clusters	Dimension	%Largest cluster
MNIST	70,000	10	784	11%
Reuters-10k	10,000	4	2000	43%
FC	10,000	4	2	50%
TM	10,000	2	2	50%
TR	10,000	3	2	50%

**Table 4:** The source domain is a dataset we can use to tune our hyperparameters. Dimension means the dimension of feature vector with source dataset. #Points means the number of data points with source dataset. The target domain is a dataset which we want to cluster using knowledge from the source.

Source Domain	Dimension	#Points	Target Domain
USPS	256	11,000	MNIST
20news	2000	$10^{4}$	Reuters-10k
(FC, TM, TR)	(2, 2, 2)	($10^{4}$, $10^{4}$, $10^{4}$)	FC
(FC, TM, TR)	(2, 2, 2)	($10^{4}$, $10^{4}$, $10^{4}$)	TM
(FC, TM, TR)	(2, 2, 2)	($10^{4}$, $10^{4}$, $10^{4}$)	TR

**Table 5:** The accuracy of estimated labels with hub data points for both MNIST and USPS is shown. The labels are estimated by SGSC. $k_{1}$ and *h* mean the number of neighbors and the number of hub data points, respectively. Each accuracy is computed by the output ${\left\{{\hat{y}}_{i}\right\}}_{i=1}^{n}$ of SGSC using corresponding the pair $\left(k_{1},h\right)$. Another number of neighbors $k_{0}$ used in the SGSC is fixed to ten. The bold font below means the best accuracy for each dataset. Please note that since we use all data points of both MNIST and USPS for the estimation, no standard deviation is shown.

Dataset		$k_{1}$	5	10	15	20	25
	*h*						
Target:	200		0.88	0.78	0.76	0.75	0.79
	300		0.45	**0.92**	0.91	0.90	0.89
**MNIST**	400		0.79	0.83	0.86	0.90	0.88
	500		0.39	0.88	0.86	0.86	0.89
Source:	200		0.59	0.49	0.51	0.44	0.46
	300		0.49	0.65	0.49	0.52	0.48
**USPS**	400		0.65	0.61	0.63	0.48	0.50
	500		0.68	**0.69**	0.64	0.65	0.53

**Table 6:** The mean accuracy and standard deviation obtained by SGSC using best hyperparameters are shown. These numbers are averaged number over seven times experiments. Since we use all samples of MNIST for the estimation, no standard deviation is shown.

MNIST	Reuters-10k	FC	TM	TR
0.88	0.78 (0.06)	0.94 (0.04)	0.98 (0.01)	0.97 (0.02)

**Table 7:** The mean clustering accuracy (ACC) of Equation (9) and standard deviation are shown. Five popular clustering methods and our proposed method were tested on five datasets. For each method, Average means the averaged ACC over the five datasets. The experiments were conducted seven times on each pair of method and dataset. For each dataset, the bold font means the highest accuracy among six methods.

Method	MNIST	Reuters-10k	FC	TM	TR	Average
k-means	0.53	0.53 (0.04)	0.60 (0.05)	0.64 (0.04)	0.35 (0.03)	0.53
SC	0.72	0.62 (0.03)	0.80 (0.04)	0.85 (0.03)	0.96 (0.03)	0.79
IMSAT	**0.98**	0.71 (0.05)	0.70 (0.04)	0.66 (0.05)	0.34 (0.01)	0.68
DEC	0.84	**0.72 (0.05)**	0.72 (0.04)	0.67 (0.03)	0.48 (0.04)	0.69
SpectralNet	0.83	0.67 (0.03)	0.79 (0.03)	0.87 (0.02)	**0.99 (0.01)**	0.83
SEDC	0.89	**0.73 (0.05)**	**0.95 (0.03)**	**0.96 (0.02)**	**0.99 (0.00)**	**0.90**

**Table 8:** The corresponding mean runtime (seconds) and standard deviation of Table 7 are shown. Five popular clustering methods and our proposed method were tested on five datasets. For each method, Average means the averaged runtimes over the five datasets. The experiments were conducted seven times on each pair of method and dataset. Please note that the runtimes of SC, IMSAT, SpectralNet, and SEDC do not include the runtimes of hyperparameter tuning. As for DEC, the runtime does not include the runtime of pre-training. The bold font means the fastest runtime among six methods.

Method	MNIST	Reuters-10k	FC	TM	TR	Average
k-means	**136**	**30 (2.3)**	**0.04 (0.0)**	**0.05 (0.0)**	**0.03 (0.0)**	**33.2**
SC	96,362	473 (5.7)	417 (4.3)	408 (5.1)	413 (4.9)	19,614
IMSAT	5097	749 (8.2)	429 (5.0)	424 (4.7)	428 (3.8)	1425
DEC	1274	258 (3.1)	121 (4.9)	135 (5.2)	153 (6.6)	388
SpectralNet	2239	232 (2.4)	122 (2.0)	110 (2.3)	117 (2.5)	564
SEDC	3466	440 (4.4)	226 (1.9)	233 (3.0)	237 (3.4)	920
